# Diversity and Biotic Homogenization of Urban Land-Snail Faunas in Relation to Habitat Types and Macroclimate in 32 Central European Cities

**DOI:** 10.1371/journal.pone.0071783

**Published:** 2013-08-06

**Authors:** Michal Horsák, Zdeňka Lososová, Tomáš Čejka, Lucie Juřičková, Milan Chytrý

**Affiliations:** 1 Department of Botany and Zoology, Masaryk University, Brno, Czech Republic, Czech Republic; 2 Department of Biology, Masaryk University, Brno, Czech Republic, Czech Republic; 3 Institute of Zoology, Slovak Academy of Sciences, Bratislava, Slovakia; 4 Department of Zoology, Charles University in Prague, Praha, Czech Republic; Roehampton university, United Kingdom

## Abstract

The effects of non-native species invasions on community diversity and biotic homogenization have been described for various taxa in urban environments, but not for land snails. Here we relate the diversity of native and non-native land-snail urban faunas to urban habitat types and macroclimate, and analyse homogenization effects of non-native species across cities and within the main urban habitat types. Land-snail species were recorded in seven 1-ha plots in 32 cities of ten countries of Central Europe and Benelux (224 plots in total). Each plot represented one urban habitat type characterized by different management and a specific disturbance regime. For each plot, we obtained January, July and mean annual temperature and annual precipitation. Snail species were classified into either native or non-native. The effects of habitat type and macroclimate on the number of native and non-native species were analysed using generalized estimating equations; the homogenization effect of non-native species based on the Jaccard similarity index and homogenization index. We recorded 67 native and 20 non-native species. Besides being more numerous, native species also had much higher beta diversity than non-natives. There were significant differences between the studied habitat types in the numbers of native and non-native species, both of which decreased from less to heavily urbanized habitats. Macroclimate was more important for the number of non-native than native species; however in both cases the effect of climate on diversity was overridden by the effect of urban habitat type. This is the first study on urban land snails documenting that non-native land-snail species significantly contribute to homogenization among whole cities, but both the homogenization and diversification effects occur when individual habitat types are compared among cities. This indicates that the spread of non-native snail species may cause biotic homogenization, but it depends on scale and habitat type.

## Introduction

The accelerating rate of urbanization in most of Europe since the 1950s has led to a dramatic increase of urban areas [1]. Urbanization process, which produces anthropogenic habitats, affects biodiversity in various ways. On one hand, large cities harbour an important component of biotic diversity [2]: they were repeatedly found to be richer in native plant species than their surrounding areas (e.g. [3,4]) and to support populations of endangered species (e.g. [5]). On the other hand, urbanization results in native habitat loss and is often considered as a major threat to native species diversity (e.g. [6,7]). Urban areas contain a greater proportion of non-native species than their surroundings (e.g. [8–10]); for example, non-native plant species comprise about 40% of the total floras of Central European cities [3] and a similar proportion in individual urban habitats [11].

The introduction of non-native (alien, exotic) species to new areas, especially if accompanied by a decline in native species, may lead to biotic homogenization, i.e. increasing similarity of species composition between different areas [12,13]. However, invasions of non-native species and extinctions of native species at some sites can also lead to community diversification [14,15], which is scale-dependent [16]. There is a growing body of evidence of various taxa homogenization, but most of the data relate to vascular plants (e.g. [7,16,17]) and vertebrates (e.g. [18,19]). Studies on invertebrates are still rare with a few exceptions; for example Blair and Launer [20] also studied butterflies, in addition to birds. There is also one study on land snails of the Pacific islands [21], but without explicit tests of the effect of non-native species on faunal composition. Molluscs, especially land snails, are known to experience the highest proportion of extinctions among the major taxonomical groups of animals [22], while many non-native and pest species have been introduced to various regions (see 23 for Europe). Although there are rather good data on urban land-snail faunas, especially in Europe (see 24 for review [25]), no study has explored the effect of non-native snails on the homogenization or diversification of snail communities in cities. No comparison has been made across urban habitat types and with no attempt to assess which habitat types support a larger diversity of native or non-native species.

Using a standardized protocol for land-snail sampling in seven urban habitat types in 32 cities of Central Europe, Belgium and the Netherlands, we studied the richness of native and non-native species in relation to urban habitat types that differed in the intensity of human management and disturbance regime. We hypothesized that (1) numbers of native species would increase and those of non-native species would decrease with decreasing disturbance intensity; (2) urban land-snail faunas would be more homogeneous due to the introduction of non-native species; and (3) the effect of non-natives on community similarity would differ between urban habitat types: the level of homogenization would be stronger in the frequently disturbed habitats under stronger human pressure.

## Methods

### Data set

**Figure 1:**
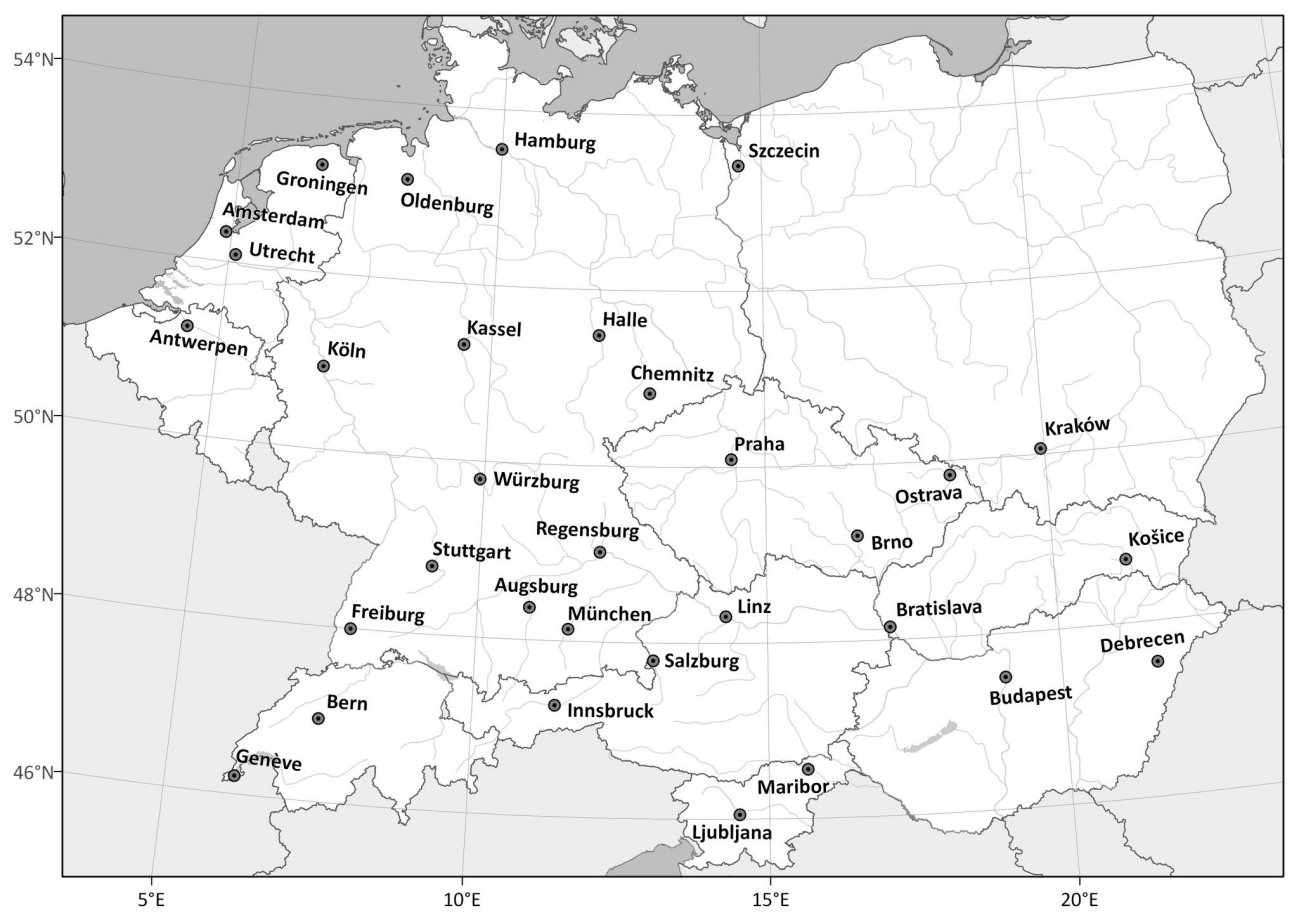
Location of the studied cities in Central Europe.

We collected land snails in 32 cities in Belgium, the Netherlands, Germany, Poland, Czech Republic, Slovakia, Switzerland, Austria, Slovenia and Hungary (Figure 1); each city had > 100,000 inhabitants (for the map of the studied cities see also 15,25). Although the study area exceeds the traditional borders of Central Europe to the northwest, for simplicity it is referred to as Central Europe throughout this paper. The choice of the studied cities was stratified based on the Central European macroclimatic pattern in order to limit correlation between climatic variables, in particular to separate the effects of temperature and precipitation on land-snail diversity. The cities were selected from ad hoc established climatic regions based on mean annual temperature (range 7.9–11.2 °C, two regions were established with temperatures lower or higher than 9 °C, respectively), January–July temperature difference (range 14.2–23.0 °C; division level 19 °C) and annual precipitation sum (range 544–1312 mm; division level 700 mm; data from [26]). Two regions based on each of these three variables yielded eight climatic regions when combined. Four cities, located as far as possible from each other, were sampled in each climatic region. The mean distance between cities within the regions was 250 km. Further details and a map of the climatic regions can be found in Lososová et al. [25].

The sampling was conducted from mid-June to late August in 2007–2009. Species composition (presence/absence) was recorded in seven 1-ha plots of square or rectangular shape (the latter in habitat patches narrower than 100 m) in each city. Each of them represented one urban habitat type: (1) historical city square; (2) boulevard with 19th century houses; (3) residential area with compact building pattern (“garden cities”); (4) residential area with open building pattern, consisting of blocks of flats built in the 1960s–1980s; (5) city park with lawns and old deciduous trees; (6) early successional sites, strongly disturbed one to three years ago, usually in or around construction sites; and (7) mid-successional sites abandoned for 5–15 years, with scattered shrubs and young trees. Detailed descriptions of these habitat types are given in Lososová et al. [25]. In each plot, all land snails were searched for by eye in all appropriate microhabitats for 1–2 hours, depending on habitat heterogeneity and species richness (see 27), with special attention paid to looking for slugs and minute species. The time spent on a plot corresponded with the proportion of paved or sealed area, i.e. less time was spent at historical city squares and boulevards than in the other habitat types. All live snail individuals, as well as empty shells with an intact periostracum, were considered. Slugs were fixed in 70% ethanol and identified based on anatomical characters in the laboratory, if necessary. Nomenclature follows Horsák et al. [28]. No legally protected species were collected or treated during the sampling. Most of the shelled species were identified directly in the field, which was always the case for the few nationally threatened (i.e. included in national red lists) species, e.g. *Granariafrumentum* . No specific permissions were needed for the sampling as we collected only on land open to the public with free access.

The species were classified according to their status as either native or non-native to each country on the basis of national lists of non-native species, national checklists of molluscs, papers on distribution of individual non-native species, and distributional atlases [29,30]. Following this up-to-date classification based on the current literature we obtained a close agreement with the DAISIE database [23]; only in 18% and 6% of cases, respectively, one or two more non-native species were recorded per plot than if the analysis was based on the DAISIE database, which does not contain some of the newer records.

### Statistical analysis

Sample-based rarefaction curves [31] were used to compare the numbers of native and non-native species across the studied cities. These curves were computed as means of 10,000 sample-based species-accumulation curves that resulted from a random ordering of species lists from all cities. This calculation was done using the JUICE program, version 7 [32].

Differences in the number of native and non-native species among the studied habitat types and the effect of climatic variables on species richness were tested using generalized estimating equations (GEE) with a Poisson error structure (GEE-p). GEE is an extension of the generalized linear models for situations when measurements of the response variable are not independent. In our study there were multiple observations made in each city, which gave rise to a correlated response per city, so we assumed exchangeable correlation structure in the residuals in order to correct for standard errors of parameter estimates that were too small. GEE provides correct marginal or population average models even when correlation structure is not perfectly specified [33]. GEE were fitted using a function from the ‘geepack’ package (version 1.1–6 [34]). The significance of all predictors was tested using Wald test. In the case of the factor for habitat type, individual levels were combined if there was no significant difference (such levels are indicated by the same letters). All analyses were performed in the R environment (version 2.15.2 [35]).

To determine if a pair of assemblages had been homogenized or differentiated due to the introduction of non-native species, we calculated the homogenization index [36]: H = J_total_ -J_native_, where J_total_ is the Jaccard similarity [37] between two sites calculated using all species and J_native_ is that based on native species only; H ranges from −1 to 1. Positive values indicate that the similarity for native species is lower than that for all species, which means that non-native species contribute to homogenization. Negative H-index values indicate that the introduction of non-native species contributes to the differentiation of species composition among assemblages. Differences in similarity between all and native species for cities and individual habitats were tested by a paired Wilcoxon non-parametrical test.

## Results

**Figure 2:**
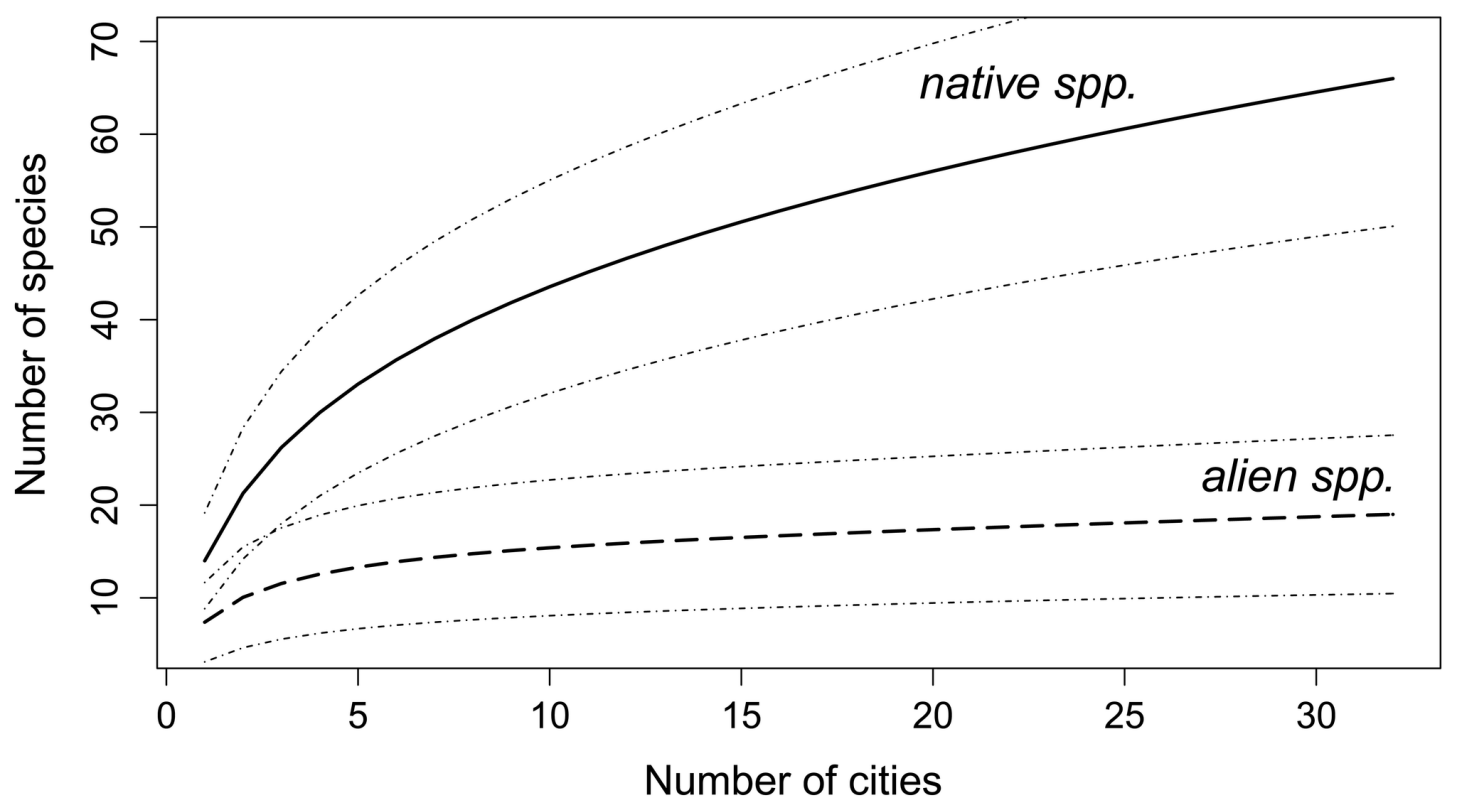
Sample-based rarefaction curves showing an increase in the cumulative number of native and alien land-snail species recorded in 32 Central European cities with increasing number of cities sampled. Dashed lines indicate 95% confidence intervals.

**Figure 3:**
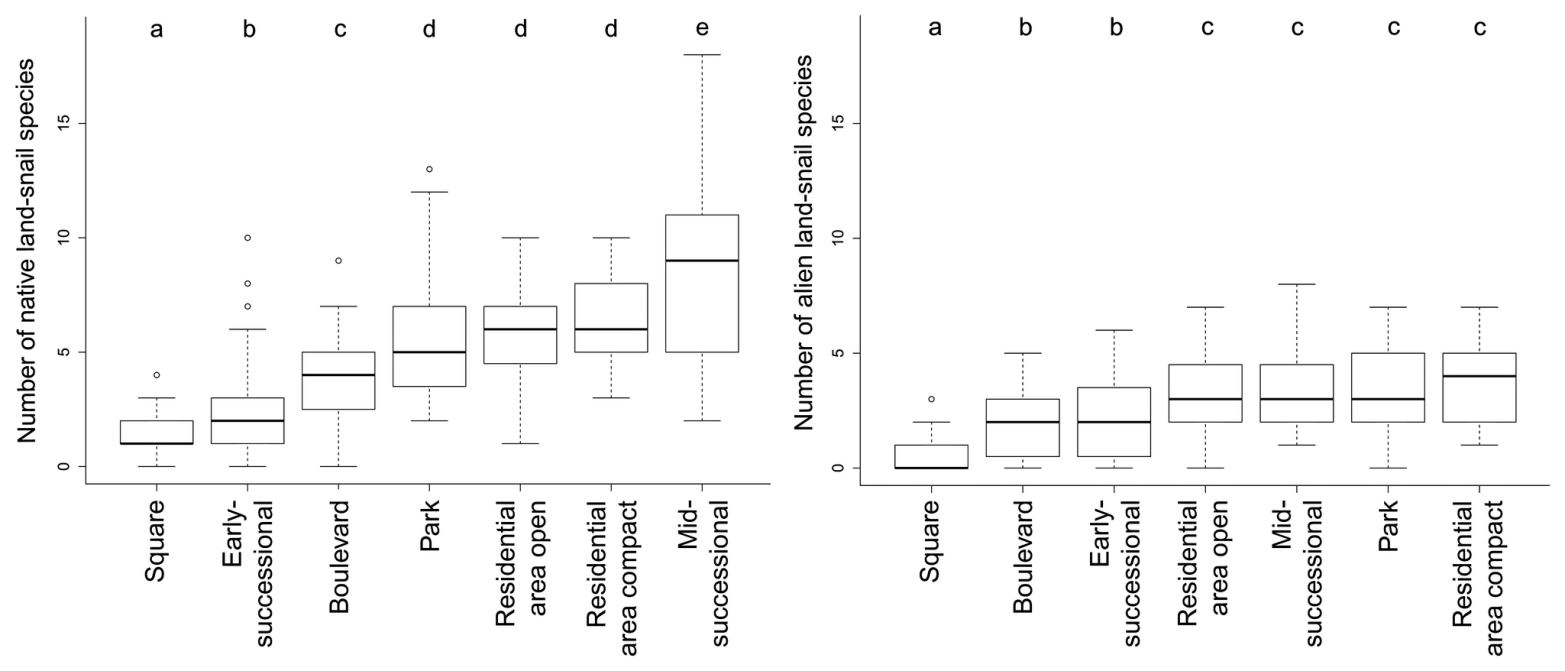
Variation in numbers of native (left) and alien (right) land-snail species among the studied habitat types. Different letters at the top indicate significant differences between habitats based on generalized estimating equations with a Poisson error structure: X^4^_2_ = 186.4, p << 0.001 for native and X^4^_2_ = 86.2, p << 0.001 for alien species; individual categories of habitats significantly differed among each other at p < 0.02. The central line of each box refers to the median value, box height to the interquartile range, whiskers to the non-outlier range (i.e. 1.5 times the interquartile range at each side), and small circles to outliers.

In total, we recorded 67 native and 20 non-native land-snail species in seven urban habitat types of 32 Central European cities (Table S1). Richness of native species per city was significantly higher than for non-native species (Figure 2). The latter also had a much lower beta diversity than the former, as their species pool was captured after only few cities had been sampled. There were significant differences between the studied urban habitat types in terms of the number of both native and non-native species (Figure 3). Unmanaged successional sites with scattered shrubs and trees were the richest for native species (median of nine species, Table 1), in contrast to historical city centres and boulevards, which harboured few native species (median of one and four, respectively). The latter two habitat types, and recently disturbed sites, were also the poorest in non-native species, unlike the other habitats that hosted significantly more non-native species (Figure 3). Native species experienced significant differences in their numbers, resulting in five groups of habitat types differing in species richness, in contrast to non-native species reaching virtually the same numbers in all habitat types but city squares. The proportion of non-native species in the whole cities was in total about 36%, being notably higher only in strongly disturbed habitats in or around construction sites (54%, Table 1).

**Table 1:** Median numbers of native and alien species and the percentage of alien species in the whole faunas of the seven urban habitat types studied.

	Native	Alien	% of alien
Square	1	0	10
Boulevard	4	2	32
Residential area closed	6	4	38
Residential area open	6	3	37
Park	5	3	36
Early successional site	2	2	54
Mid-successional site	9	3	25

We found no effect of climatic variables on native species richness, except for annual temperature having a significant negative effect (GEE-p, *χ*_*1*_^2^ X^2^_1_ = 5.3, p = 0.022). In contrast, both continentality (GEE-p, X^2^_1_ = 4.8, p = 0.028) and annual precipitation (GEE-p, X^2^_1_ = 4.3, p = 0.038) were found to have a significant effect on the number of non-native species, which slightly increased towards more humid conditions and more rapidly declined towards cities with a smaller difference between the mean temperature in January and July.

**Figure 4:**
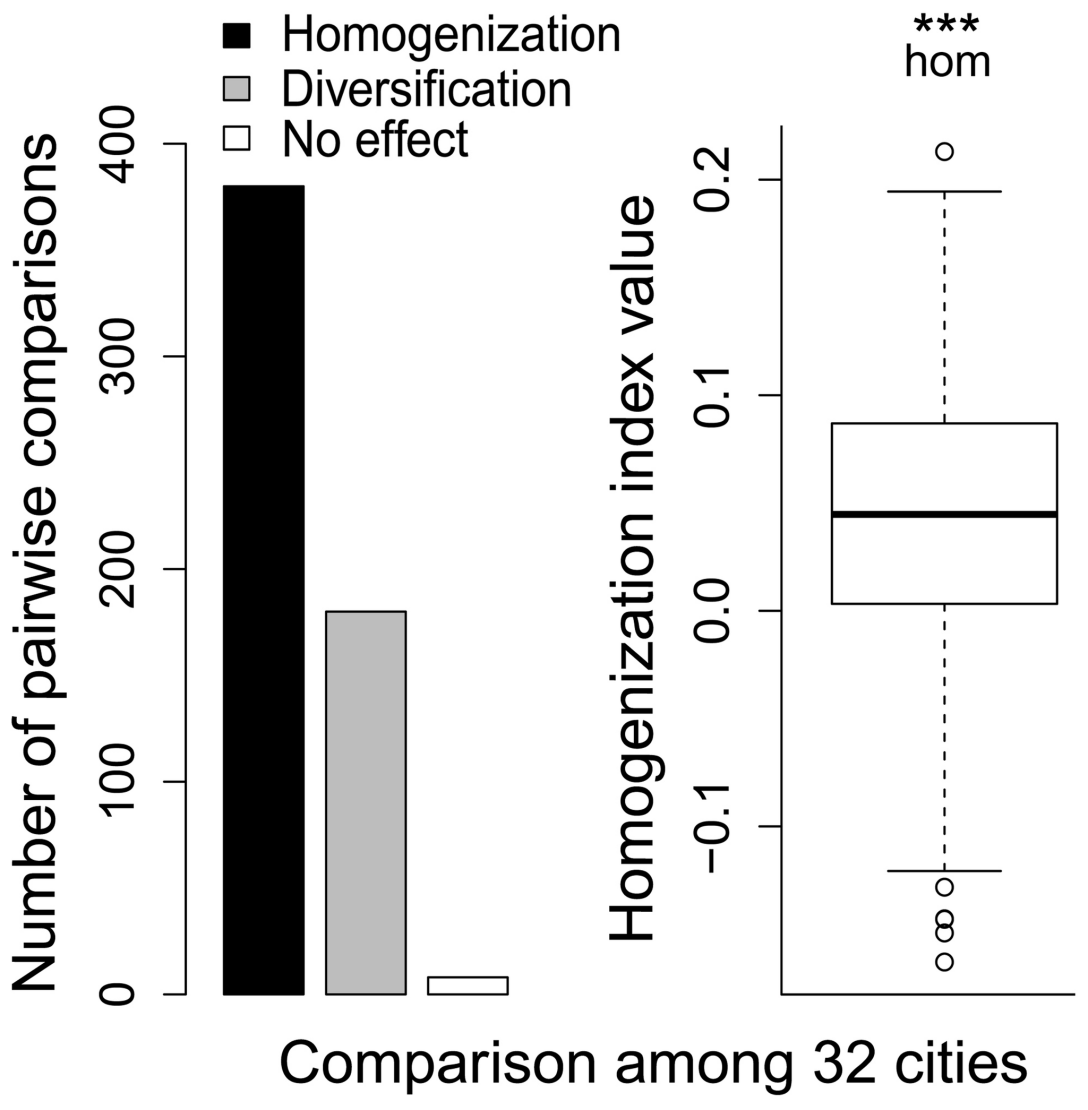
Numbers of positive (homogenization) and negative (diversification) values of the homogenization index resulting from pairwise comparisons among 32 cities (left), and variation in values of the homogenization index among the studied habitat types, showing the homogenization (hom) effect of alien species on species composition similarity. Differences between Jaccard similarities based on all and native species were tested using a paired Wilcoxon test (***, p < 0.001). For explanation of box-and-whisker plots see Figure 3.

**Figure 5:**
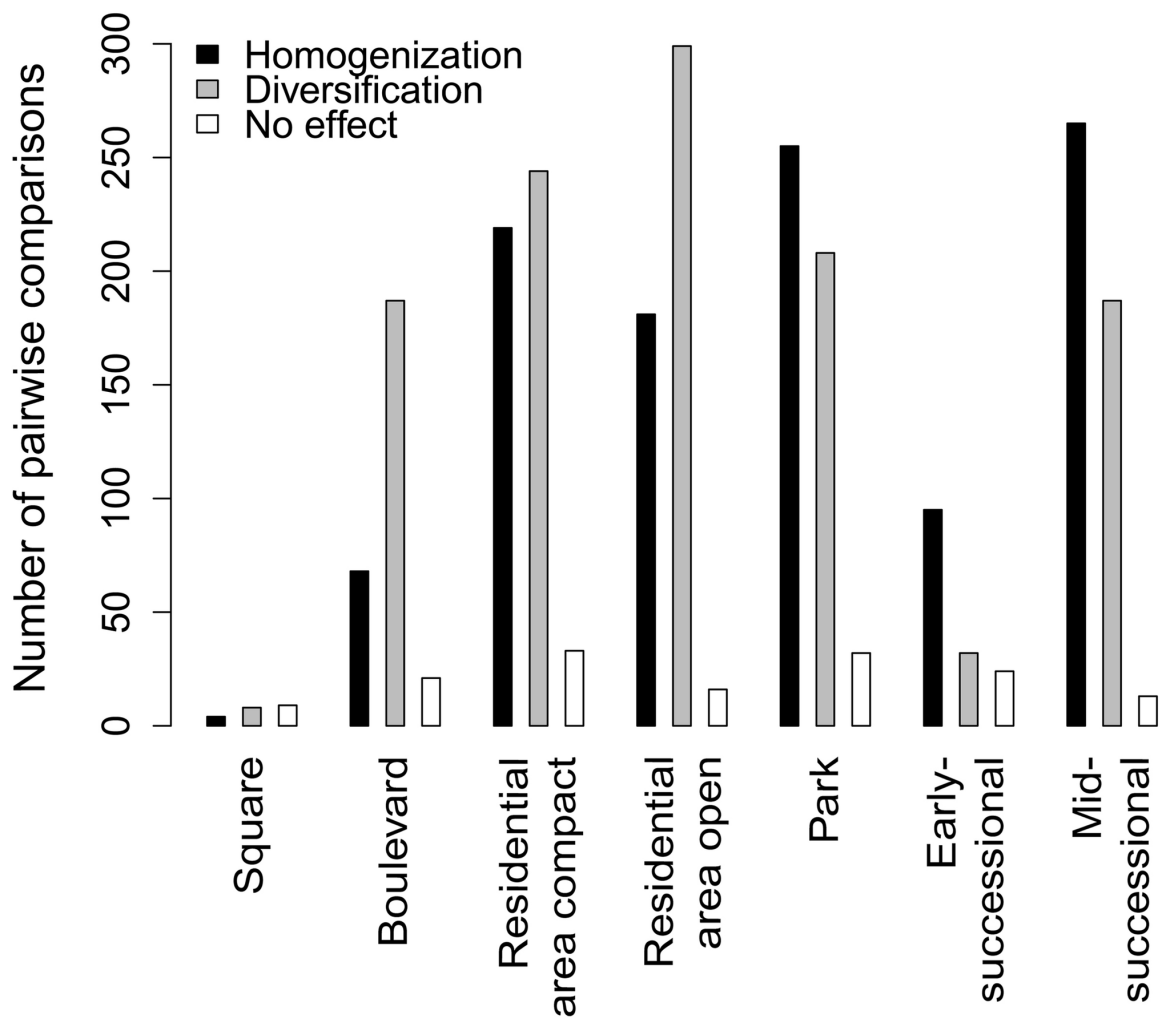
Numbers of positive (homogenization) and negative (diversification) values of the homogenization index resulting from pairwise comparisons among 32 cities and calculated separately for seven types of urban habitats. Only those plots that harboured four or more species were used, which implies a different number of comparisons for each habitat type: square (4% of all 496 possible pairwise comparisons were considered), boulevard (56%), residential area with open building pattern (100%), residential area with compact building pattern (100%), park (94%), early-successional site (30%), mid-successional site (94%).

**Figure 6:**
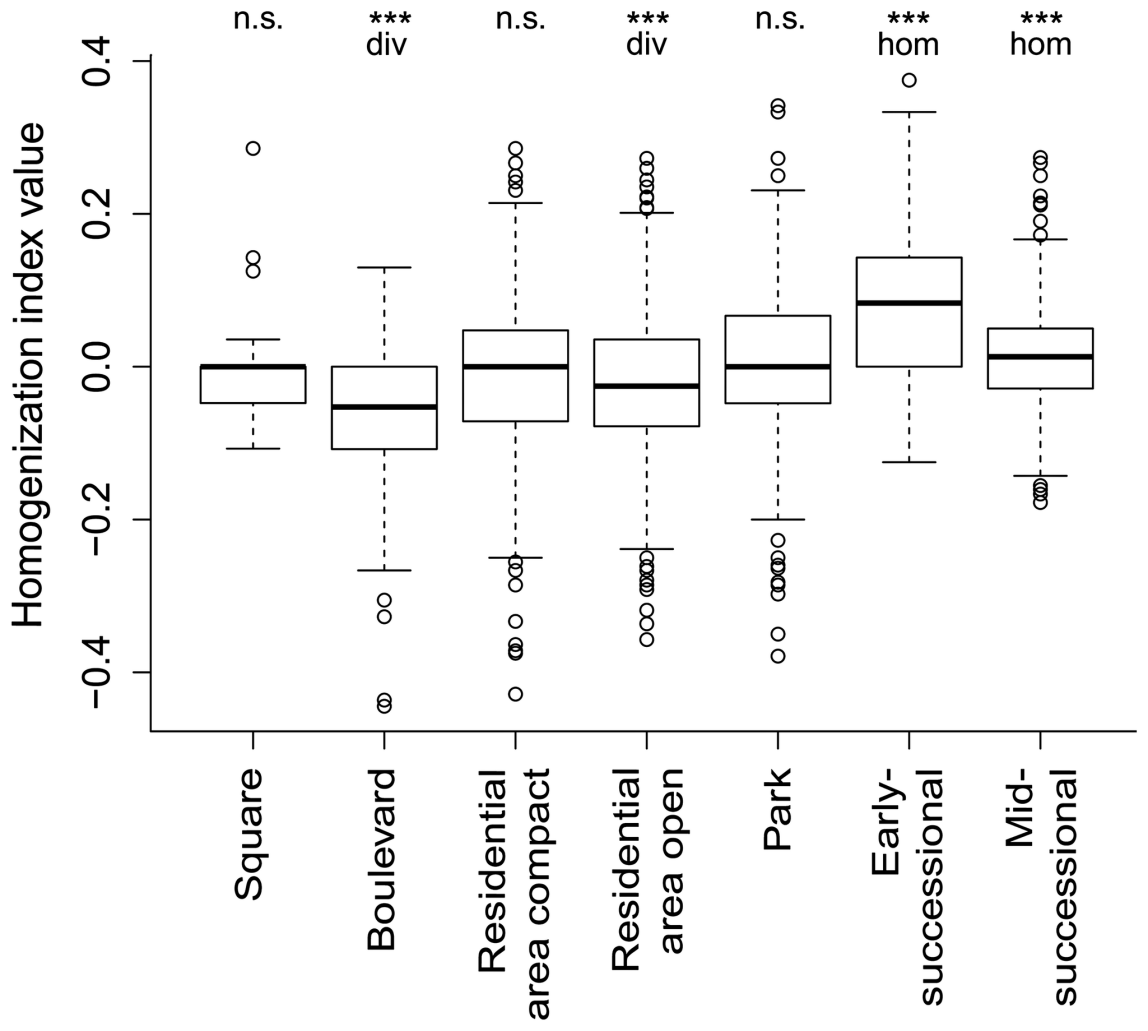
Variation in the values of homogenization index among the studied habitat types showing the diversification (div) or homogenization (hom) effect of alien species on species composition similarity. Differences between Jaccard similarities based on all and native species were tested using paired Wilcoxon test; significance: **, p < 0.01; ***, p < 0.001; n.s., not significant. div = diversification, hom = homogenization. For numbers of pairwise comparisons for each habitat type see Figure 5.

Considering the whole cities, we found a significantly higher homogenization than differentiation effect of non-native species on community composition (Figure 4). However, these effects were not uniform across the urban habitat types (Figures 5 and 6). We found significant homogenization caused by non-native species at unmanaged successional sites with scattered shrubs and trees and, even more pronounced, at recently disturbed sites. In contrast, the presence of non-native species resulted in diversification of species composition in boulevards and residential areas with an open building pattern (Figures 5 and 6).

## Discussion

### Diversity and the effect of climate

The number of non-native species in large regions is known to be controlled by macroclimate, especially temperature and precipitation [38]. For cities and urban habitats, macroclimatic relationships have been studied especially for plants [3,15,25,39,40]. As far as we know, however, such studies are lacking for urban land-snails, both in Central Europe and elsewhere. In this study we found that the number of non-native land-snail species significantly increased with increasing amount of annual precipitation, which was mostly due to the marked increase in slug species (their richness was analysed in a separate model, data not shown). As slugs are in general more sensitive to desiccation, they favour humid conditions (e.g. [41]). Non-native species richness also increased towards areas with a lower January–July temperature difference. As many non-native species originate from southern regions with mild winters (e.g. *Cornu aspersum*, *Eobania* *vermiculata* , *Hygromiacinctella* and *Tandonia* *budapestensis* ), they are favoured by the suboceanic and oceanic climate of Western-Central and Northwestern Europe. In contrast, the number of native species did significantly and negatively respond to increasing mean annual temperature. This result is rather unexpected because it is well known that most land-snail species are favoured by a warmer climate (e.g. [42]), and many do not produce cryoprotective chemicals [43]. The response is probably too complex and requires a deeper analysis; however it can be linked with the nature of the majority of native species living in urban habitats. The most frequently recorded species are those which have survived glacial conditions in Central Europe [44], and thus their distribution is likely to be weakly affected by low temperature. This can be further linked with a higher potential invasiveness of temperate species on tropical islands [45], as these species evolved to withstand greater climatic and seasonal fluctuations.

We also observed the previously reported pattern of land-snail species richness in cities increasing from highly to less urbanized habitats [24]. This pattern has also been demonstrated for plants and various animal taxa (e.g. [20,25,46]). We found that both native and non-native species richness increased along the urbanization gradient, with highly urbanized city squares being generally extremely poor in snails. These areas harboured only a few species passively spreading with garden soil (e.g. *Vallonia pulchella* and *Derocerasinvadens* ), which lived only in flowerpots there. Our results expanded previously published observations based on the data pooled across all the habitats of individual cities, indicating that differences in the management and disturbance regime of urban habitats override the importance of climatic factors [25]. We revealed similar responses of both native and non-native species; however, management regime was more important for the diversity of native species. This has important conservation implications, as areas of favourable management for snails can host an important proportion of urban land-snail diversity and support relatively species-rich faunas, including some threatened species.

### Biotic homogenization among cities and urban habitat types

The results confirmed our first hypothesis that the land-snail faunas of Central European cities are homogenized due to the spread of non-native species. In contrast, vascular plant assemblages, studied in the same plots as the snails in this study, were found to be diversified due to the establishment of non-native species [15]. This observation has broad support in other studies conducted on floras in Central Europe (e.g. [9]) and North America (e.g. [7]). It has previously been reported that different introduction dynamics among taxa are related to differences in their introduction patterns [47]. For plants, the diversity of introduced non-native species is strongly correlated with human population density, as many of them are introduced for horticultural or agricultural purposes (e.g. [38,48]). This is one of the reasons why cities tend to have a higher total numbers of plant species that their surroundings [3]. The proportion between non-native species invasion and extirpation of rare native species, related or unrelated to invasions, determines the diversification or homogenization among communities, with a resulting pattern that is strongly scale- and time-dependent [9,16]. Homogenization is more likely to occur across larger spatial extents [49] and seems to be a truly global phenomenon [13]. In contrast, on smaller, local or regional scales several mechanisms linked to urbanization promote the establishment of more heterogeneous assemblages [50]. The resulting pattern can be viewed in a spatial or temporal context as shown by Kühn and Klotz [9] for Central European flora. Many of the plant species introduced before 1500 A.D. (archaeophytes) have had sufficient time to occupy suitable habitats across larger spatial extents (e.g. [4]). Therefore their assemblages are rather similar across large spatial scales [51] and they significantly contribute to homogenization [15]. In contrast, it has been repeatedly shown that species with a shorter residence time such as non-native species introduced after 1500 A.D. (neophytes) are responsible for between-community diversification (e.g. [9,15]). The opposite result that was recorded for snails compared to plants [15] in the studied 32 Central European cities seems to result from a small number of non-native snail species, of which only a few have spread recently. However, one of them, *Arion vulgaris* (syn. *A* *. lusitanicus* ), the most frequent non-native species in our dataset (Table S1), has spread extremely fast over the last 60 years. The shape of the rarefaction curve for the non-native snails in our data set (Figure 2) and that of archaeophytes recorded in the same plots ( [15]: Figure 2) was very similar: they both flattened out after only five cities had been sampled. This suggests that non-native land snails as a group behaved in the same way as the pre-1500 non-native plants, showing a similar pattern of their distribution among studied cities.

Although we found a prevailing effect of homogenization among the land-snail faunas of Central European cities if data from different plots within cities were combined, there were important differences among urban habitat types (Figures 3 and 4), with a strong homogenization found at early-successional sites exposed to the highest level of disturbance. This finding fits with our previous observation for vascular plants, for which the most pronounced homogenization was found for the archaeophytes recorded in this habitat type [15]. This suggests that both archaeophytes and non-native land snails in urban environments include common species that are well-adapted to disturbances. Many of these species (all land-snail species found in more than 10 plots; Table S1) were more frequent in urbanized areas that elsewhere, and can therefore be termed “urban specialists” [52]. In contrast, a significant diversification effect of non-native snail species was found in boulevards and residential areas with an open building pattern; in the latter, the same pattern was revealed for vascular plant assemblages [15]. In contrast to plants, non-native snail faunas of boulevards mostly contributed to diversification, probably due to the very low numbers of species recorded and relatively high importance of the stochastic occurrence of some non-native species.

To conclude, using a standardized sampling in seven urban habitat types in 32 Central European cities, we documented the prevailing homogenization effect of non-native land-snail species on urban snail faunas. The number of non-native species significantly increased towards more humid climates, but for both native and non-native species the effect of climate on diversity was much less pronounced than the effect of urban habitat types, with their specific management and disturbance regimes. Although in two habitat types the presence of non-native species caused diversification and in another two it resulted in homogenization, the only pronounced effect was the biotic homogenization observed at highly disturbed early-successional plots. This suggests that the effect of non-native species on biota homogenization is not universal: it depends on scale and habitat types.

## Supporting Information

Table S1 **List of native** and **alien species recorded in 32 Central European cities; numbers of plots** and **cities with the species presence are given.**
(PDF)
