# Supplementary material for: Diversity and Biotic Homogenization of Urban Land-Snail Faunas in Relation to Habitat Types and Macroclimate in 32 Central European Cities
Source: PLoS One. 2013 Aug 6;8(8):e71783. doi: 10.1371/journal.pone.0071783 (PMC3735557; doi:10.1371/journal.pone.0071783)
Supplement: Table S1 — List of native and alien species recorded in 32 Central European cities; numbers of plots and cities with the species presence are given. (PDF) [file pone.0071783.s001.pdf]

Table S1. List of native and alien species recorded in 32 Central European cities; numbers of plots and cities with the species presence are given.

**Native species**	No. of plots	No. of cities
Cochlicopa lubrica (Müller, 1774)	131	32
Vallonia pulchella (Müller, 1774)	120	28
Trochulus hispidus (Linné, 1758)	91	26
Vallonia costata (Müller, 1774)	84	24
Cepaea nemoralis (Linné, 1758)	72	18
Helix pomatia Linné, 1758	68	22
Cepaea hortensis (Müller, 1774)	66	21
Discus rotundatus (Müller, 1774)	62	23
Aegopinella nitidula (Draparnaud, 1805)	41	17
Alinda biplicata (Montagu, 1803)	35	15
Pupilla muscorum (Linné, 1758)	27	12
Arianta arbustorum (Linné, 1758)	22	11
Vitrina pellucida (Müller, 1774)	20	16
Xerolenta obvia (Menke, 1828)	18	12
Zonitoides nitidus (Müller, 1774)	18	13
Monachoides incarnatus (Müller, 1774)	16	12
Aegopinella minor (Stabile, 1864)	15	10
Oxychilus cellarius (Müller, 1774)	15	7
Cepaea vindobonensis (Férussac, 1821)	11	6
Perpolita hammonis (Strøm, 1765)	11	7
Fruticicola fruticum (Müller, 1774)	10	10
Succinea putris (Linné, 1758)	10	8
Succinella oblonga (Draparnaud, 1801)	10	10
Oxychilus alliarius (Müller, 1774)	9	3
Arion circumscriptus Johnston, 1828	8	5
Arion silvaticus Lohmander, 1937	8	5
Deroceras laeve (Müller, 1774)	8	7
Vertigo pygmaea (Draparnaud, 1801)	8	6
Arion intermedius Normand, 1852	5	3
Cecilioides acicula (Müller, 1774)	5	5
Urticicola umbrosus (Pfeiffer, 1828)	5	5
Candidula intersecta (Poiret, 1801)	4	2
Helicella itala (Linné, 1758)	4	2
Merdigera obscura (Müller, 1774)	4	3
Candidula unifasciata (Poiret, 1801)	3	2
Cochlodina laminata (Montagu, 1803)	3	3
Deroceras agreste (Linné, 1758)	3	2
Euomphalia strigella (Draparnaud, 1801)	3	3
Helix lutescens Rossmässler, 1837	3	1
Laciniaria plicata (Draparnaud, 1801)	3	3
Trochulus sericeus (Draparnaud, 1801)	3	2
Carychium tridentatum (Risso, 1826)	2	2
Clausilia parvula Férussac, 1708	2	1
Helicodonta obvoluta (Müller, 1774)	2	2

Table S1. Continuation.

	No. of plots	No. of cities
Lauria cylindracea (Da Costa, 1778)	2	1
Limax cinereoniger Wolf, 1803	2	2
Punctum pygmaeum (Draparnaud, 1801)	2	2
Truncatellina cylindrica (Férussac, 1807)	2	1
Aegopinella nitens (Michaud, 1831)	1	1
Arion fuscus (Müller, 1774)	1	1
Carychium minimum Müller, 1774	1	1
Clausilia bidentata (Strøm, 1765)	1	1
Cochlicopa lubricella (Rossmässler, 1835)	1	1
Ena montana (Draparnaud, 1801)	1	1
Granaria frumentum (Draparnaud, 1801)	1	1
Helicigona lapicida (Linné, 1758)	1	1
Macrogastra plicatula (Draparnaud, 1801)	1	1
Oxychilus glaber (Rossmässler, 1835)	1	1
Perforatella bidentata (Gmelim, 1791)	1	1
Plicuteria lubomirskii (Ślósarskii, 1881)	1	1
Pseudotrichia rubiginosa (Rossmässler, 1838)	1	1
Semilimax semilimax (Férussac, 1802)	1	1
Tandonia rustica (Millet, 1843)	1	1
Truncatellina callicratis (Scacchi, 1833)	1	1
Vitrea crystallina (Müller, 1774)	1	1
Vitrea subrimata (Reinhardt, 1871)	1	1
Vitrinobrachium breve (Férussac, 1821)	1	1
**Non-native species**		
Arion vulgaris (Moquin-Tandon, 1855)	101	29
Deroceras reticulatum (Müller, 1774)	93	29
Oxychilus draparnaudi (Beck, 1837)	74	28
Arion distinctus Mabille, 1868	65	25
Deroceras invadens (Reise, Hutchinson, Schunack et Schlitt, 2011)	43	15
Limax maximus Linné, 1758	43	23
Deroceras sturanyi (Simroth, 1894)	33	15
Monacha cartusiana (Müller, 1774)	33	19
Hygromia cinctella (Draparnaud, 1801)	25	7
Cornu aspersum (Müller, 1774)	18	5
Arion fasciatus (Nilsson, 1823)	17	13
Boettgerilla pallens Simroth, 1912	11	9
Tandonia budapestensis (Hazay, 1881)	11	8
Zonitoides arboreus (Say, 1816)	7	7
Limacus flavus (Linné, 1758)	2	1
Paralaoma servilis (Shuttleworth, 1852)	2	2
Candidula intersecta (Poiret, 1801)	1	1
Cecilioides petitiana (Benoit, 1862)	1	1
Eobania vermiculata (Müller, 1774)	1	1
Helix cincta Müller, 1774	1	1
Hygromia limbata (Draparnaud, 1805)	1	1
